# Proof of Concept of a New Revision Procedure for Ceramic Inlays of Acetabular Cups Using a Shape-Memory Alloy Actuator System

**DOI:** 10.3390/bioengineering11090868

**Published:** 2024-08-27

**Authors:** Christian Rotsch, Karoline Kemter-Esser, Johanna Dohndorf, Kerstin Funke, Christoph-Eckhard Heyde, Welf-Guntram Drossel

**Affiliations:** 1 Fraunhofer Institute for Machine Tools and Forming Technology IWU, 01187 Dresden, Germanyjohanna.dohndorf@tu-dresden.de (J.D.); kerstin.funke@iwu.fraunhofer.de (K.F.); welf-guntram.drossel@iwu.fraunhofer.de (W.-G.D.); 2 Department of Orthopaedic, Trauma and Plastic Surgery, University of Leipzig Medical Center, 04103 Leipzig, Germany; christoph-eckhard.heyde@medizin.uni-leipzig.de; 3 Institute of Biomedical Engineering, Faculty of Electrical and Computer Engineering, TUD Dresden University of Technology, 01307 Dresden, Germany; 4 Professorship Adaptronics and Lightweight Design, Faculty of Mechanical Engineering, Chemnitz University of Technology, 09107 Chemnitz, Germany

**Keywords:** hip arthroplasty, implant design, modular endoprosthesis, revision surgery, acetabular cup, shape-memory alloy, SMA, NiTi, nitinol, actuator design

## Abstract

The revision of ceramic inlays of acetabular cups is a challenging surgical procedure. The mechanical impact during the inlay extraction process can damage the ceramic or metal cup rim. To avoid these risks, a concept for a new revision procedure was developed. It is based on an actuator system, which allows a non-destructive release of the ceramic inlay. To integrate the actuator system, different design concepts of acetabular cup components were investigated, and an actuator based on shape-memory alloy (SMA) wires was developed. The process chain for the actuator, starting from nickel-titanium wires manufactured into the actuator geometry by laser welding and thermo-mechanical treatment for the shape setting process up to the functionality evaluation of the actuator system, was implemented on a laboratory scale. The new revision procedure is based on a phase transformation of the SMA wire actuator, which was obtained through two methods—applying an electrical current by an instrument and rinsing the wire with heated water. The phase transformation of the actuator resulted in a contraction between 3.2% and 4.3% compared to its length after pre-stretching and was able to release the ceramic inlay from the cup. Therefore, the developed actuator design and process chain is a proof of concept towards a new revision procedure for modular acetabular cups.

## 1. Introduction

The application of ceramic bearing components in hip arthroplasty is a well-established practice due to the excellent wear properties and biocompatibility of the material [1,2,3,4]. However, releasing ceramic inlays of modular acetabular cups remains a challenge in the revision of implants and implant components due to the conical clamping. Polyethylene inlays can be removed intraoperatively by drilling and extracting or by extracting with an expanding instrument.

In comparison, the time required for removing ceramic inlays can be significantly longer. Furthermore, the risk of damaging the inlay or metal rim of the cup increases due to the mechanical stress during the revision process. Methods for releasing a ceramic inlay can include, for example, the application of a mechanical vibration, an impulse to the edge of the cup utilizing tools such as hammer and chisel, or the use special instruments followed by removing the ceramic inlay once the conical press connection has been loosened [5,6,7]. Another invasive technique is drilling into the metal cup rim to create a contact point for an impactor to release the inlay [8]. These revisions procedures can result in ceramic fragments and particles remaining in the joint even after intense cleaning of the field.

An internal actuator could be an alternative to the use of an additional external mechanical device to loosen the ceramic inlay. Previous attempts of using actuators to integrate additional functionality in hip implant components have focused on sensory functionality, for example, measuring implant forces [9], monitoring the implant bone ingrowth status [10,11], or influencing the press fit on the implant bone interface [12].

Most studies focused on the hip stem. One aspect to consider is the space required by the additional components. In comparison to a hip stem, an acetabular cup has a thinner wall strength, which limits the possibilities of integrating additional components. If an actuator component was to be integrated in an acetabular cup to release the ceramic inlay, it should have a high energy density. Compared to other conventional actuator mechanisms, e.g., electromagnetic or electrostatic actuators, shape-memory alloy (SMA) actuators based on nickel-titanium (NiTi) have the highest specific actuation energy of up to 10^3^ J/kg and energy densities up to 10 J/cm^3^ [13,14]. Therefore, very compact actuator designs and integration strategies are possible.

Commonly the one-way effect is used which is based on a diffusionless shear in the crystal lattice. This effect is caused by the phase transformation between a low-temperature phase (martensite) and a high-temperature phase (austenite). Even after an apparent plastic deformation, the initial geometry can be restored by heating the material. Since the last couple of decades, SMAs have been used for different actuator solutions in industrial, automotive, and medical applications [13,15,16,17,18,19].

In addition to the manufacturing of NiTi by additive manufacturing technologies [20,21,22], the use of NiTi wires, springs, and sheets [16,23,24] remains an interesting alternative for various applications. The processing of these materials is well established [25]. While there are numerous challenges associated with process parameters, laser welding is a well-established procedure for NiTi, particularly for NiTi wires [26,27,28,29,30].

For shape setting, usually a thermomechanical treatment of the actuator devices is necessary. The process parameters depend on the phase transition temperatures and the alloy composition. Especially for medical applications, a finishing process of the surface is necessary to remove released particles and the oxide layer [31,32,33,34,35].

This study demonstrates the incorporation of novel techniques for extracting ceramic inlays using shape-memory alloys. To this end, an actuator based on laser-welded SMA wires was developed to exert a force on the ceramic inlay. This approach could facilitate the development of a revised concept for implant components.

## 2. Materials and Methods

### 2.1. Concepts for Fixing and Releasing Ceramic Inlays Using SMA Components

The initial phase of the development process entailed a concept study. The objective was to devise concepts for securing and releasing the interface between a ceramic inlay and a metal cup. The primary goal was to minimize the risk of damaging the cup components during revision surgery. The approaches are based on the utilization of shape-memory alloys, which exhibit pseudo-elasticity (super-elasticity) or pseudo-plasticity (thermally activable) properties due to their differing crystal structures (martensitic or austenitic). Table 1 shows the resulting concepts which can be differentiated as follows:

(A) Using the conventional conical press connection of the components with new releasing mechanisms (Concepts 1–5).
(B) Complete reconfiguration of the geometrical interface of cup and inlay to create a modified mechanical connection that can be used as an alternative or supplement to conical clamping (Concepts 6 and 7).

Category (A) maintains the standard conical press connection and therefore the intraoperative joining process of the components. This approach is expected to have higher clinical acceptance. Concepts 2–4 are based on the thermal activation of the SMA to retract the inlay from the cup by pushing or pulling, whereas Concept 5 solely uses the pseudoelastic effect of the SMA by preloading and fixating the actuator with screws. When the SMA clamp is released, the inlay is pushed out of the cup. Concept 1 is similar to Concept 5 by preloading pseudoelastic SMA clamps, but in this case the clamps are restrained by thermally activated SMA.

Category (B) examines new geometrical approaches to combine a ceramic inlay and a hip cup. Concept 6 establishes a screw connection, which is secured by a thermally activated SMA component, whereas Concept 7 uses pseudoelastic SMA elements functioning as a click-connection by holding the inlay in position. Due to the new mechanical connections a new surgical procedure is needed to implant the ceramic inlays described by Concepts 6 and 7.

### 2.2. Clinical and Technical Requirements for the Final Implant and Actuator Design

The different concepts were analyzed according to clinical and technical requirements, e.g., minimal change of geometry, retaining the original functionality and interfaces, mechanical stability, and biocompatibility of materials used. The hip cup should be modified as little as possible, both in terms of internal and external geometry, so that the basic surgical procedure and surgical instruments require only minor changes. The following materials are used as basis:

- “Multicup II” metal cup made of a titanium–alumina–vanadium alloy (TiAl6V4 Grade 5) from Aristotech Industries GmbH, Luckenwalde, Germany,
- “ceramys” ceramic inlay from Mathys Orthopädie GmbH, Mörsdorf, Germany.

To release the ceramic inlay from the acetabular cup, a pushout actuator force of 6 kN was defined. This value is based on measurements of dynamic loads on implant components conducted by Mathys Orthopädie GmbH, which considered the effects of various forces and loads on the ceramic inlay due to movement, stumbling, and possible tilting including safety factor. The results are in the same range as in vivo measurements with instrumented implants [9].

The risk analysis of the developed concepts (Table 1) has shown that Concepts 6 and 7 of Category (B) result in significant changes of the functionality of the implant components. Beside adapting designs and production processes, new tests and standards would be required to guarantee a safe implant. In some cases, extensive changes to the cup and ceramic inlay would be necessary. Clinicians have also expressed concerns about the introduction of a completely new implant concept. Therefore, concepts of Category (A) were prioritized in this study (Table 1, Concepts 1–5).

Due to the restriction in space and the high manufacturing effort, Concepts 1 and 5 were discarded. The remaining concepts (2–4) were theoretically designed regarding the requirements of the SMA elements. The efficiency (force and stroke) of the SMA elements depends on the force direction in relation to the crystal lattice structure. Contracting actuators have a bigger stroke than push-actuators and therefore need less design space [19]. This resulted in eliminating Concept 4. Concepts 2 and 3 are based on the same functionality. Concept 3 requires less construction space, as the inlay is directly pushed out without an additional element. Table 2 shows the ranking of the concepts.

We chose a wire actuator to fulfil the described requirements for Concept 3. The actuator in the cup area was designed with a maximum stress of 200 MPa to 800 MPa, a maximum change in length of 3% to 8%, and an assumed uniaxial stress state. An actuator arrangement can achieve the necessary pushout forces due to the attainable stresses of 630 N to 2500 N per section (see Table 3). However, this is a simplified consideration for the actuator geometry and low activation cycles.

We assumed a maximum actuator stress of 600 MPa and a maximum elongation of 6% for the final design.

**Figure 1:**
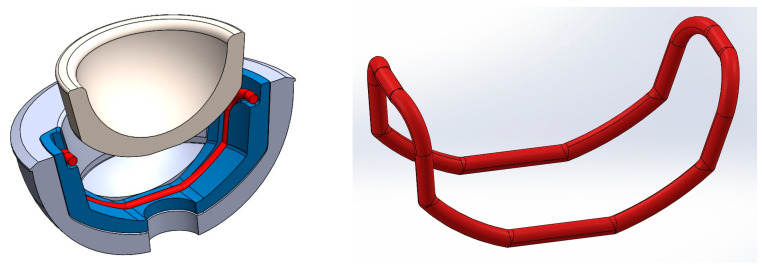
CAD model of actuator (red), peek-inlay (blue), ceramic inlay (white), and metal cup (light blue): sectional view (**left**) and SMA actuator (**right**).

Figure 1 shows the final design of the new acetabular cup concept. It consists of a modified ceramic inlay “ceramys” 32/E (Mathys Orthopädie GmbH, Mörsdorf, Germany), a modified “Multicup II D58” metal cup made of a titanium–alumina–vanadium alloy (TiAl6V4 Grade 5), a polymer inlay, and the SMA actuator. Pursuing Concept 3 (Table 1), the actuator loop shortens due to thermal activation, thereby releasing the inlay from the press fit.

### 2.3. Inlay Components—Materials and Experimental Setup

The inlay provided thermal insulation and mechanical retention for the actuator wire. Therefore, it must withstand the actuator forces without deforming during activation. Polyoxymethylene (POM), chirulen/ultra-high molecular weight polyethylene (UHMW-PE), and polyether ether ketone (PEEK) were considered as options, being stable in shape, biocompatible, and commonly used in medical technology [36,37,38,39].

**Figure 2:**
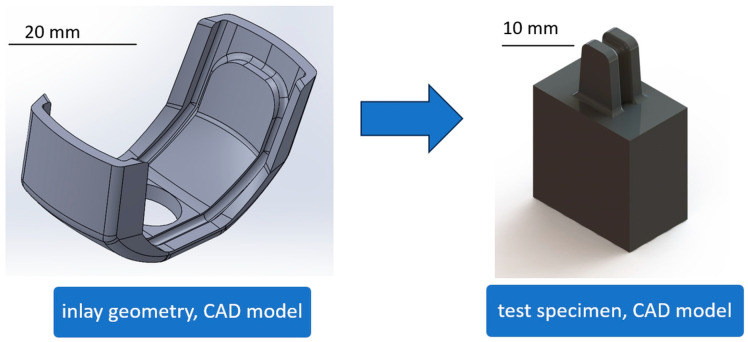
Polymer inlay geometry and derived geometry for the material deformation analyzation.

To ascertain the suitability of the materials as inlay component, test samples were developed for load investigations. The geometry was based on the design of the inlay and represents a worst-case scenario in terms of bending radii (Figure 2).

The test specimens of POM, UHMW-PE, and PEEK (Table 4) were manufactured by Aristotech Industries GmbH, Luckenwalde, Germany. The deflection points for the SMA wire (diameter 2 mm) are represented in the sample components.

**Figure 3:**
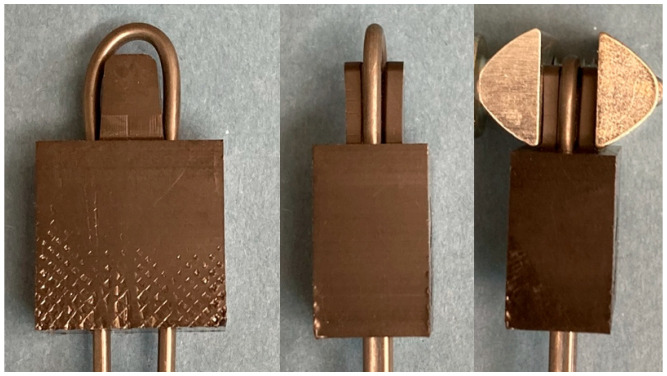
PEEK sample with SMA wire (**left**, **middle**) and with additional clamping element (**right**) after material deformation test procedure.

**Figure 4:**
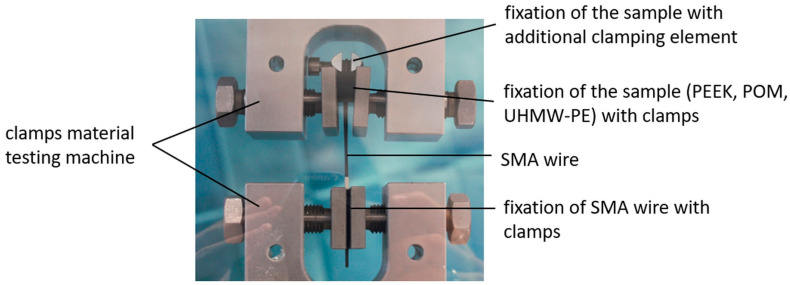
Experimental set up for material characterization of the inlay components.

As part of the tensile experiments, the components underwent mechanical loading using a tensile–compression–torsion testing machine (DYNA-MESS Prüfsysteme GmbH, Aachen, Germany). The actuator wire was stretched over the test specimen and it was fixated by the upper clamp. To prevent the sample components from being bent open by the wire, a screw clamp was also pressed around the upper narrow point of the test specimen (Figure 3, right). The wire ends were then secured in the lower clamp (Figure 4), which can be moved downwards to apply the tensile force. The distance resulting from the deformation of the polymer and the potential elongation of the SMA wire was quantified. This allows force–distance behavior to be evaluated instead of Young’s modulus of the polymer specimens. The tensile force was increased in 500 N increments (at a speed of 50 N/s) with a holding time of 3 s after every 500 N increase until a final force of 3500 N was reached or the material was destroyed. A force of 3500 N was assumed to be the maximum force occurring in the real load case, distributed over two sides of the actuator. Furthermore, a safety factor of 1000 N was assumed in total, with 500 N on each side.

### 2.4. Shape-Memory Alloy Actuator—Manufacturing Process and Experimental Setup

For our study we used a Nickel–Titanium–Copper–Chromium (NiTiCuCr) wire with a diameter of 1.95 ± 0.015 mm (Ni42.5Ti49.9Cu7.5Cr0.1 alloy, Ingpuls GmbH, Bochum, Germany). The NiTiCuCr alloy as base material had an austenitic finish temperature of 52 °C, which was confirmed by differential scanning calorimetry (DSC) (STARe System DSC, Mettler-Toledo GmbH, Gießen, Germany). DSC analysis was also conducted on the finished actuators to analyze the influence of the subsequent joining and heat treatment processes.

The actuator was a closed SMA wire loop that was joined using laser welding (Trumpf TruDisk 10002, Trumpf, Ditzingen, Germany). Test rigs were designed to join wires with a length between 130 mm and 134 mm. The joint underwent high mechanical stress during the molding process and must be capable of absorbing and transferring actuator forces. A study was conducted to find the final laser parameters (focus diameter: 0.4 mm, power: 1300 W, pulse duration: 100 ms; shielding gas: argon).

**Figure 5:**
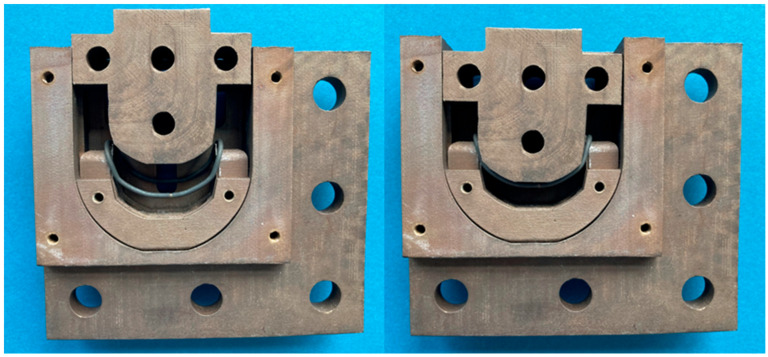
Forming tool with SMA actuator without cover: open position (**left**), closed position (**right**).

In the following process step, the final actuator geometry was molded using the tool displayed in Figure 5. First, the SMA wire loop was pressed into the defined wire guide using a punch by using a tensile–compression–torsion testing machine (DYNA-MESS Prüfsysteme GmbH, Aachen, Germany). A maximum force of 1400 N with a speed of 0.25 mm/s was applied three times, unloading after the first two load cycles. After applying the load the third time, the wire was fixed in the tool and then heat treated in a muffle furnace (Nabatherm Model N 15/65 A, Nabatherm, Lilienthal, Germany) at 500 °C for 30 min, followed by quenching in a water bath (room temperature, approximately 22 °C) to imprint the target geometry in the austenitic state. For fast heating and cooling, the mold had several holes to reduce its mass and thermal capacity. The mold also permitted the wire actuators to be pre-stretched. Therefore, a force up to 1600 N with a speed of 0.25 mm/s was applied.

**Figure 6:**
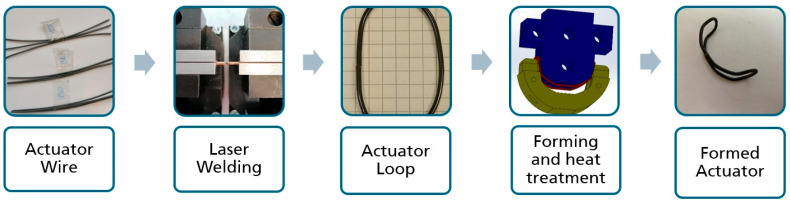
Process of actuator manufacturing.

Figure 6 shows the complete process sequence from actuator wire to final actuator geometry.

**Figure 7:**
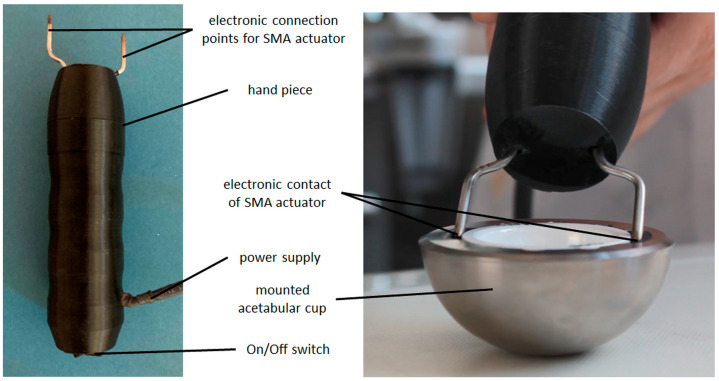
Activation instrument: overview (**left**), in contact with mounted SMA actuators (**right**).

Two methods were compared for thermal activation:

(1) Activation by placing the actuators in tempered water (70–80 °C) for up to 1 min as reference for complete phase transformation.
(2) Activation using electrical resistance heating by applying an activation instrument provided by endocon GmbH, Wiesenbach, Germany, to the mounted actuators (see Figure 7, right).

To evaluate the functionality of the actuators, studies were conducted to join and release the ceramic inlays. The actuators were pre-stretched and positioned in the cup with the peek inlay. The ceramic inlay was placed and then joined with a tensile–compression–torsion testing machine (DYNA-MESS Prüfsysteme GmbH, Aachen, Germany) with a joining force of 2000 N and a speed of 0.04 mm/s in accordance with ASTM F1820 [40]. Then the actuator was heated by the activation instrument, releasing the ceramic inlay.

As reference, only the SMA actuator was placed in heated water after the pre-stretching, because with this approach a complete phase transformation can be assumed.

The two different activation methods were compared using an optical measuring system and associated software (ZEISS GOM scanner, ZEISS GOM inspect software 2022 SP3, Carl Zeiss GOM Metrology GmbH, Braunschweig, Germany) to record changes in length and geometry in the pre-stretched, electrically activated, and water-activated states.

Subsequently, µCT examinations (Phoenix V|tome|x S240, Waygate Technologies, Wunstorf, Germany) were employed to visualize the components in both the joined and activated/released states, examining their change in length or position. The activation was achieved by placing the entire implant in heated water. The geometry and positional changes were analyzed using the software VGSTUDIO MAX 2024.2, (Volume Graphics GmbH, Heidelberg, Germany).

## 3. Results

### 3.1. Inlay Components—Experimental Results

**Figure 8:**
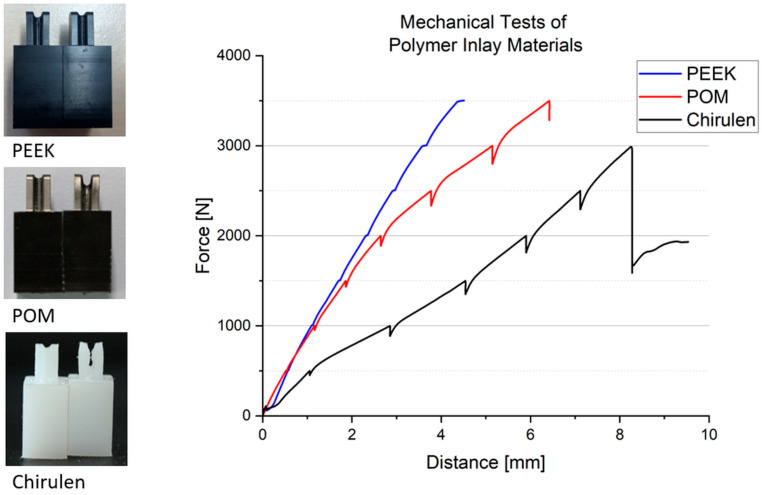
Mechanical experiments of support materials.

Figure 8 displays the deformation test results, indicating that the POM material was suitable for forces up to 3500 N. However, a flattening of the curve was apparent as the force increases, which can be explained by a slight yielding of the material. Furthermore, the process of material settlement during holding times was evidenced by a reduction in force (about 50 N to 300 N depending on the applied force). A comparison of the sample before and after the experiment revealed a cut into the material (see Figure 8, left).

Chirulen should not be subjected to forces exceeding 3000 N. The material experiences a uniform increase in force initially, but at 3000 N the wire cut into the sample, causing material failure. This was evident when comparing the samples before and after the experiments (see Figure 8, left).

The PEEK material could withstand forces of up to 3500 N. A uniform increase in force was seen. The increase was the highest compared to POM and Chirulen, which means that the material yielding was the lowest. Minimal discontinuities in the force–distance curve occur during the holding time. With a nearly constant force applied, the distance increased minimally (about 0.05 to 0.15 mm). The material could withstand the applied force. The result of this investigation was that PEEK was the most suitable material for use in the metal cup. An additional six PEEK samples were evaluated for a comparative study. The result was confirmed and incision depths in the material between 0.2–0.7 mm (Table 5) were observed. These did not appear to be critical for functionality due to their depth.

### 3.2. Shape-Memory Alloy Actuator—Experimental Results

**Figure 9:**
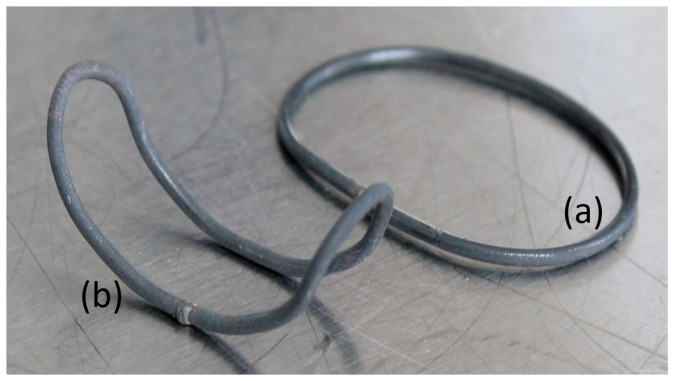
SMA actuator: laser welded (**a**) and formed (**b**).

Figure 9 shows the SMA actuator after laser welding to form a wire loop (a) and molding in the tool depicted in Figure 5 to reach the final actuator geometry (b).

The actuators and the weld seam could not withstand the impact force during shape setting and pre-stretching in all cases. Nevertheless, in a published study regarding push-out forces [41] we were able to demonstrate the reliable functioning of selected actuators.

**Figure 10:**
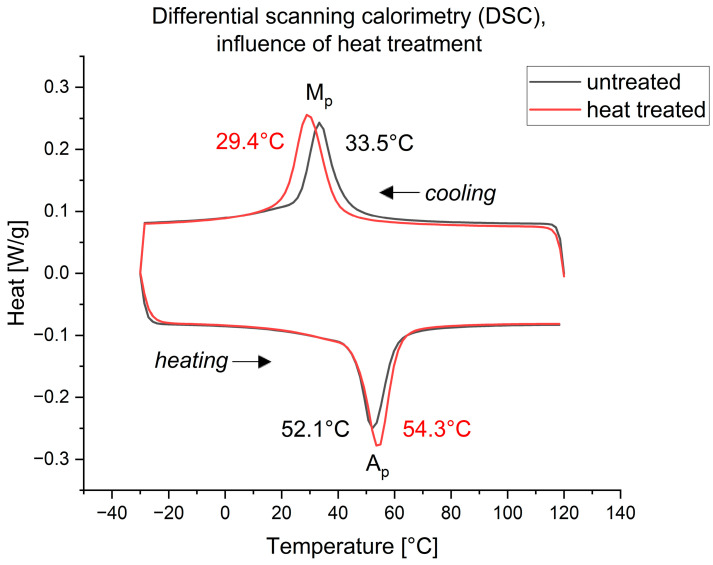
Differential scanning calorimetry (DSC) of NiTiCuCr alloy for the determination of the phase transformation temperatures: untreated wire (black) and after welding and heat treatment (red). The peaks of the heat difference depending on temperature show the phase transformations of the alloy. The upper graph shows the cooling procedure, the lower graph the heating process.

The exemplary DSC analysis shown in Figure 10 indicates a slight shift in transformation temperatures between the starting material and the final actuator. Specifically, the martensite peak temperature (M_P_) shifted from 33.5 °C to 29.4 °C, while the austenite peak temperature (A_P_) shifted from 52.1 °C to 54.3 °C.

**Figure 11:**
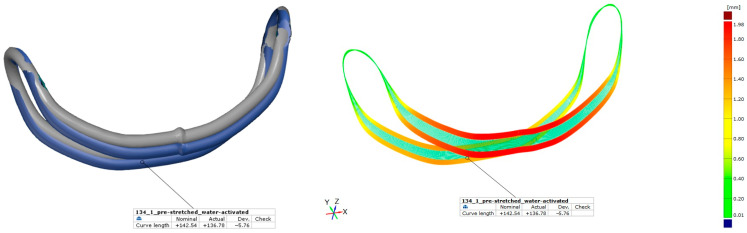
Comparison of measured curve geometries of actuator 134_1: pre-stretched vs. water-activated. Surface geometry, blue—pre-stretched, grey—water-activated (**left**) and distance between geometries (**right**).

The phase transition (activation) of the actuators could be achieved successfully using electrical heat resistance and tempered water. The greatest difference between the activated and pre-stretched wires could be achieved using tempered water [41]. The results of the optical analyzation are presented in Table 6 and exemplified in Figure 11 for actuator 134_1. The difference in length of the actuator is clearly visible. Figure 11 on the right shows the difference between the geometries of both actuator conditions.

**Figure 12:**
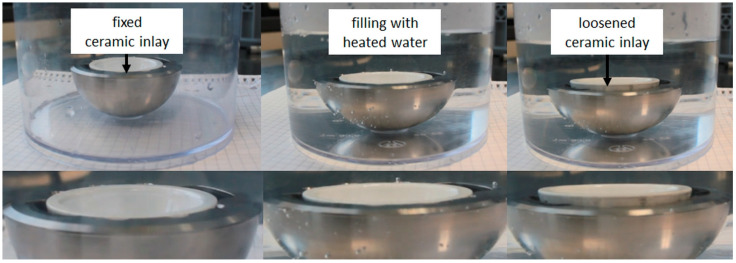
Activation of SMA actuator and releasing of ceramic inlay by using heated water.

By placing the mounted acetabular cup in heated water, the actuator contracted and released the ceramic inlay which was mounted according to ASTM F1820 [40] (Figure 12).

**Figure 13:**
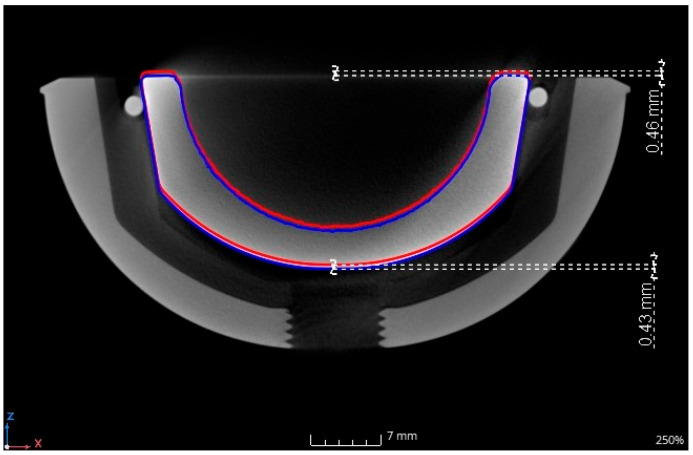
µCT image of initial (blue line) and released (red line) ceramic inlay: sectional view. Example visualization with actuator wire 134_2.

Figure 13 shows the fixed and released ceramic inlay as µCT image. The inlay components are shown in the initial situation. The actuator wire (134_2) was pre-stretched and the ceramic inlay was mounted according to ASTM F1820 [40]. The blue line shows the contour of the ceramic inlay. The red line shows the contour after activation with heated water visualized with a second µCT scan. Both images were merged. The position of the ceramic inlay after activation (red line) and the initial position (blue line) is shown for comparison. The translation in the z-direction was measured at two points: at the bottom and at the top edge of the ceramic inlay in relation to the acetabular cup. The change in the relative position of the acetabular cup and the ceramic inlay is clearly visible and measured at 0.43 mm and 0.46 mm. The difference was the result of a minimal dumping of the ceramic inlay during assembly.

**Figure 14:**
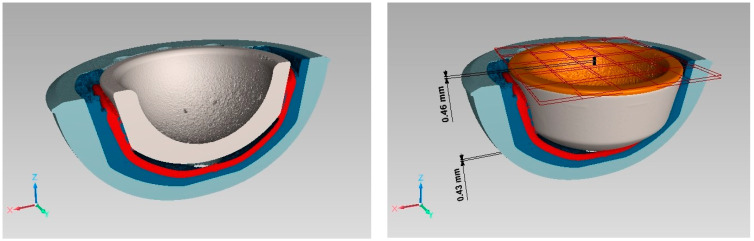
3D–µCT image of implant components: sectional view at initial state (**left**) and ceramic inlay position at initial state and after activation (**right**). Colors: grey—ceramic inlay at initial position; orange—ceramic inlay after activation; red—actuator device; blue—PEEK inlay; light blue—metal acetabular cup, red grids to visualize the top of the inlay at different positions.

Figure 14 shows the implant components and the shifting of the ceramic inlay after activation of actuator 134_2 as a 3D image.

## 4. Discussion

### 4.1. Implant Design and Inlay Components

The aim of this study was the development of a design concept for an acetabular cup for a novel procedure to facilitate the removal of ceramic inlays. Of the seven concepts presented, Concept 3 was deemed the most suitable in terms of installation space and modifications to the standard acetabular cups. To achieve this, an acetabular cup (“Multicup II D58”) was modified by adapting the inner geometry and integrating an additional polymer inlay between the ceramic inlay (“ceramys” 32/E) and metal cup. These modifications allowed the integration of an SMA actuator device and a standard ceramic inlay. The conventional conical press connection of the components can be employed in conjunction with the developed release mechanism.

Mechanical characterization experiments revealed that a polymer inlay made of PEEK was the most suitable option compared to the materials POM and chirulen. The mechanical behavior met the expectations of the manufacturer’s specifications, as shown in Table 4. Investigations were conducted on PEEK test specimens, which could be loaded with a force of up to 3500 N. This resulted in 0.4 ± 0.2 mm deformation or incision on the test samples. The used geometry of the specimen was the worst-case scenario, because the final polymer inlay design had a higher radius of 4 mm around the deflection points. Therefore, a lower material stress was assumed. Given its biocompatibility and long-term use in implant applications, this material seemed to be applicable in further developments of the implant concept [38,42].

### 4.2. Shape-Memory Alloy Actuator

The technical feasibility of an SMA actuator was demonstrated by welding an actuator wire (NiTiCuCr) alloy into a loop and subsequently shaping and heat treating it. The resulting geometries were found to be compatible with the additional PEEK inlay and the modified acetabular cup.

It was ascertained that the welded SMA wire actuator was principally suitable for the proof of concept. Under lab conditions, the actuator was able to release a ceramic inlay after mounting the implant components according to ASTM F1820 [40]. The study did not consider dynamic loads such as those occurring during the service life of the implants, for example, due to stumbling or running [43]. These influences should be investigated in further studies.

However, not all actuators were able to withstand the mechanical stress during molding or activation. This resulted in the formation of a crack near the weld seam. Consequently, further investigations into the joining of the SMA wires are necessary. It is known that the material properties are changed by the heat input around the fusion zone and the heat-affected zone. Typically, there is a reduction in tensile strength and elongation [28,31]. Further studies should therefore investigate, for example, the energy input, focal diameter, or subsequent heat treatments.

It was observed that the heat treatment had an influence on the phase transformation temperatures. This phenomenon of shifting the phase transformation temperature of NiTi alloys is a well-known process and described in the literature [44,45,46]. The impact of these findings is estimated to be minimal for the focused application.

A variety of actuator lengths (nominally between 130 und 134 mm) was realized and the changes in shape resulting from thermal treatment were quantified using optical measurement methods. Activation was conducted using heated water and resistance heating with an activation instrument. The length of the actuators was found to change by between 3.2% and 4.3%, which corresponds to the desired maximum elongation of 6% necessarily used for the design of SMA wire actuators [16,47]. The global change in length was recorded to measure the elongation. Figure 11 (right) shows the local deformations of the actuator. As anticipated, the greatest strains are observed in the lower area (red areas). The smallest strains occur near the deflection points (upper area). However, these were subjected to the greatest tensile stress during the molding process. Since the actuator was designed for only few cycles, there was no concern in the development process. Whether this can lead to premature material fatigue must be examined in future studies.

The safety risk of a short time heating of the actuator to the phase transformation temperature of approx. 54 °C can be estimated as acceptable according to El-Khasef and Wever et al. [48,49]. The application of heated water appeared to be a more efficient method of actuation than electric current activation with the instrument. A maximum elongation of 0.2% was observed in comparison to water activation. This could be a result of an inadequate electric connection to the actuator. It is also important to note that the activation process was stopped when the inlay was removed. It is not necessarily required that the phase transformation was completed at this point of the heating process. Nevertheless, it is recommended that the activation process is conducted using heated water, as this method is more conducive to further development. However, it should be noted that the use of heated water could be a minor safety risk during a surgical procedure.

We investigated the influence of activation methods on the achievable actuator forces in a further study. Actuator forces of up to 1951 N were achieved with water activation and up to 1013 N with resistance heating [41].

The actuator design was intended to provide a push-out force of 6 kN. This aspect was not investigated in this study. The functionality of the SMA actuators was only demonstrated for ceramic inlays joined at 2 kN according to ASTM F1820 [40] under lab conditions.

### 4.3. Revision Procedure

**Figure 15:**
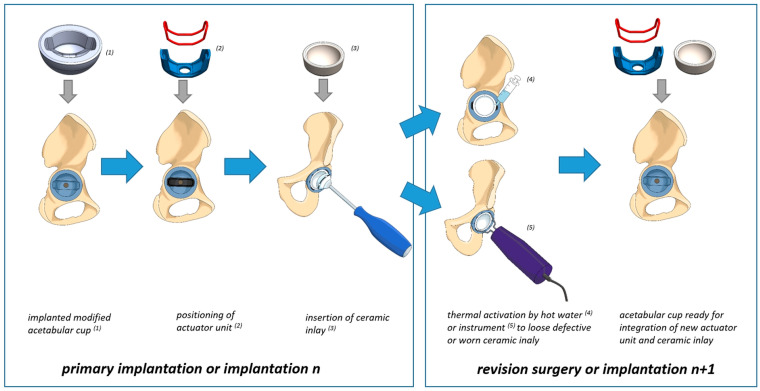
Concept of the new revision procedure for modular acetabular cups.

Based on the proof of feasibility of the SMA actuator in combination with a modified acetabular cup design we conceptionally developed a new revision procedure for ceramic inlays. It aims at replacing the ceramic inlay without damaging the acetabular cup during replacement. Figure 15 illustrates the procedure concept. During the initial implantation, the surgeon inserts the acetabular cup with the new inner design. Subsequently, the PEEK inlay and the pre-stretched SMA actuator are inserted manually without need for a special instrument. Once these components are positioned, the ceramic inlay is placed manually or with a special suction cup instrument [50]. Then, the ceramic inlay can be fixed with a standard impact instrument using a hammer. Alternatively, an automated setting instrument that exerts a defined force impulse or vibration for the inlay or the metal cup can be used [51,52]. Therefore, reproducible mechanical conditions can be realized. A new impact instrument for ceramic inlays was developed by the authors’ research group as part of a further study and its applicability was demonstrated in a laboratory environment [53].

If the ceramic terminally requires replacement due to wear or other damage, only the SMA actuator needs to be heated during the surgical procedure. This can be preformed by heated rinsing water via syringe application or by electrical heating current with an activation instrument. The ceramic inlay could be released and removed, followed by the PEEK inlay and the actuator. If the acetabular cup can still be used (sufficient osseous anchorage, no damage), a new PEEK inlay, SMA actuator, and inlay could be inserted. In the event that the metal cup must also be removed, the release procedure of the ceramic inlay can still be employed.

Our technical concept for a modified acetabular cup design and a new revision procedure ensures that no mechanical forces are transferred to the cup or the implant–bone interface during releasing. Therefore, the risk of damaging the ceramic inlay or the metal cup can be reduced. This is an advantage compared to the conventional methods applying a mechanical impact on the edge of the metal cup or other additional mechanical procedures on the acetabular cup or the ceramic inlay [5,6,7,8,50]. Additional techniques for the removal of ceramic inlays include the utilization of a suction device in conjunction with manual tapping or the employment of an acetabular trial shell as a tool in the revision process [54]. These procedures are not standardized, and the outcome is significantly influenced by the surgeon’s expertise and experience. In comparison, the approach described in our study may be easier and more reproducible to apply. Nevertheless, additional research is required to ascertain the functionality of the system, particularly with regard to the release of the ceramic inlay with the actuator system following exposure to dynamic and elevated loadings.

## 5. Conclusions

During the revision surgery of modular acetabular cup implants, it is challenging to release the ceramic insert without damaging the bone or implant components. This study presented a new design concept for acetabular cups using an integrated actuator system based on a shape-memory alloy wire to release ceramic inlays with a lower risk of damage. Therefore, different concepts were developed and analyzed. Subsequently, functional analyzations were conducted as proof of concept for the working principle. It was demonstrated that the SMA wire can be laser welded and molded into a loop with a molding tool and a thermal treatment process. Following a pre-stretching procedure, the wire actuator contracted between 3.2% and 4.3% by heating with electrical current applied by an instrument or with heated water. Under lab conditions, it was demonstrated that an inlay mounted according to ASTM F1820 [40] can be removed using the SMA actuator. Based on these laboratory results, further steps towards facilitating the revision process of ceramic inlays from acetabular cups are possible. This can reduce the mechanical impact on the ceramic insert and acetabular cup during revision, minimizing the risk of surgery complications.

## Figures and Tables

**Table 1:** Overview of design concepts. Green elements symbolize pseudoelastic SMA, red elements stand for thermally activated SMA. Concepts 1–5 are based on releasing the standard conical press connection between ceramic inlay and hip cup and Concepts 6–7 present new geometrical designs for alternative mechanical connections.

Concept Number/Category	Sketch—Sectional View	Description	Interface; Release Mechanism
1/(A)	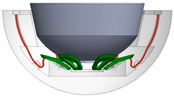	preloaded pseudoelastic SMA clamps restrained by thermally activated SMA wires; by cooling the SMA wires, the clamps are released and push out the ceramic inlay	pressfit, conical clamping; actuators must be cooled to release mechanism
2/(A)	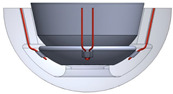	thermally activated SMA wires contracted by applying thermal energy causing them to move a ring and push out the inlay	pressfit, conical clamping; additional heat energy must be applied to release the mechanism
3/(A)	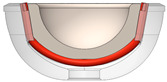	thermally activated SMA wires contracted by applying thermal energy and directly pulling out the ceramic inlay	pressfit, conical clamping; additional heat energy must be applied to release the mechanism
4/(A)	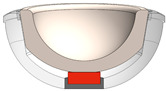	thermally activated SMA element pushes out the inlay due to heating by thermal energy	pressfit, conical clamping; additional heat energy must be applied to release the mechanism
5/(A)	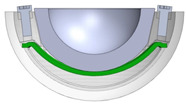	preloaded super-elastic spring element pushes out the inlay by releasing screws	pressfit, conical clamping; additional tool required for releasing the screws
6/(B)	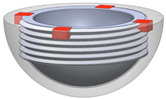	threaded inlay is blocked by thermally activated SMA elements; by cooling and removing the SMA elements, the inlay can be unscrewed from the cup	screw connection replaces conical clamping; actuators must be cooled to unblock connection
7/(B)	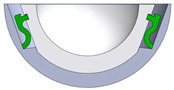	super-elastic SMA clamps lock into a recess position and fixate the inlay	pseudoelastic SMA element replaces conical clamping; additional tool required to remove SMA clamps

**Table 2:** Ranking of the design concepts for fixing and releasing ceramic inlays using SMA components.

Concept Number/Category	Geometry ^1^	Surgical Procedure ^2^	Actuator ^3^	Fail Safe ^4^	Points
1/(A)	0	1	2	1	4
2/(A)	0	1	2	1	4
3/(A)	2	2	2	1	7
4/(A)	2	2	1	1	6
5/(A)	1	1	1	0	3
6/(B)	0	0	1	0	1
7/(B)	0	0	2	0	2

^1^ change of design/geometry (minimal: 2, medium: 1, significant: 0). ^2^ change of surgical procedure (minimal: 2, medium: 1, significant: 0). ^3^ actuator (pull: 2, push: 1). ^4^ fail safe at malfunction of actuator (yes: 1, no: 0).

**Table 3:** Parameter final design concept.

Parameter	
pushout force actuator	6000 N
wire diameter	1.95–2.05 mm
wire length	50–140 mm
actuator tension	200–800 MPa
actuator strain	3–8%
actuator force	630–2500 N

**Table 4:** Polymer materials for test specimens, overview.

Material	Product Name, Manufacturer	Young’s Modulus (Tension) *
polyoxymethylene (POM)	TECAFORM AH black, Ensinger GmbH, Nufringen, Germany	2800 MPa
chirulen/ultra-high molecular weight polyethylene (UHMW-PE)	CHIRULEN^®^ 1050, Quadrant PHS Deutschland GmbH, Vreden, Germany	approx. 700 MPa
polyether ether ketone (PEEK)	TECAPEEK MT black, Ensinger GmbH, Nufringen, Germany	4200 MPa

* Manufacturer’s information.

**Table 5:** Deformation of the PEEK inlay caused by the SMA wire during the material experiments.

Incision Depth of PEEK Sample [mm]	
Sample	
1	0.3
2	0.2
3	0.7
4	0.5
5	0.4
6	0.3
mean value	0.4
standard deviation	0.2

**Table 6:** Actuator length, examples.

Probe *	Condition	Length [mm]	Strain ** [%]
130_1	pre-stretched	138.83	3.4
	current-activated	134.42	0.1
	pre-stretched	138.56	3.2
	water-activated	134.24	-
130_4	pre-stretched	139.39	4.3
	current-activated	134.25	0.4
	pre-stretched	139.22	4.2
	water-activated	133.67	-
132_5	pre-stretched	140.84	3.7
	water-activated	135.85	-
134_1	pre-stretched	142.54	4.2
	water-activated	136.78	-

* Nomenclature of the examples: [initial length of the actuator wire in mm (130 to 134)]_[consecutive number within an actuator length (1 to 5)]. ** strain relative to “water-activated”.

## Data Availability

The data presented in this study are available on request from the corresponding author.
